# Supplementary material for: The interactome of intact mitochondria by cross-linking mass spectrometry provides evidence for coexisting respiratory supercomplexes
Source: Mol Cell Proteomics. 2017 Dec 8;17(2):216–32. doi: 10.1074/mcp.RA117.000470 (PMC5795388; doi:10.1074/mcp.RA117.000470)
Supplement: Supplemental Data [file supp_RA117.000470_133922_0_supp_23978_fzffwf.zip › spectra_annotation/mito_DR_spectra_annotation/33-2-6-1-8-1.pdf]

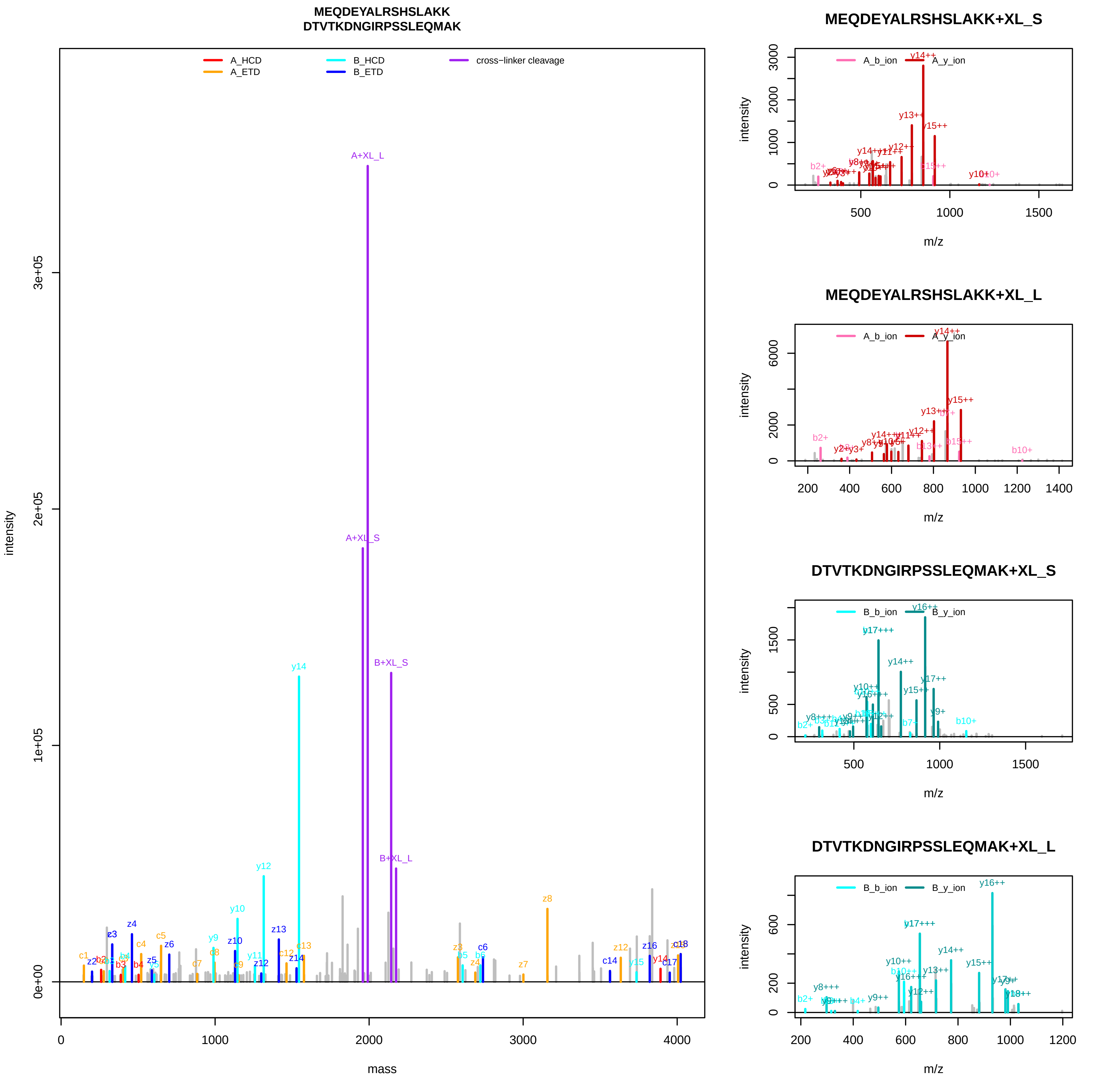
MEQDEYALRSHSLAKK
DTVTKDNGIRPSSLEQMAK
A_HCD
A_ETD
B_HCD
B_ETD
cross−linker cleavage
intensity
mass
0e+00
1e+05
2e+05
3e+05
0
1000
2000
3000
4000
A+XL_L
A+XL_S
B+XL_S
B+XL_L
y14
y12
y10
y9
y11
z4
z3
z13
z10
z6
z5
z2
z12
z14
z16
z8
z7
z3
z4
z12
c1
c4
c5
c7
c8
c9
c12
c13
c6
c14
c17
c18
b2
b3
b4
y14
y15
b5
b6
MEQDEYALRSHSLAKK+XL_S
MEQDEYALRSHSLAKK+XL_L
DTVTKDNGIRPSSLEQMAK+XL_S
DTVTKDNGIRPSSLEQMAK+XL_L
A_b_ion
A_y_ion
B_b_ion
B_y_ion
intensity
m/z
y14++
y13++
y15++
y16++
y17+++
y14++
y15++
y17++
y10++
b10+
b2+
